# Oxytocin Removes Estrous Female vs. Male Preference of Virgin Male Rats: Mediation of the Supraoptic Nucleus Via Olfactory Bulbs

**DOI:** 10.3389/fncel.2017.00327

**Published:** 2017-10-23

**Authors:** Xiao-Yu Liu, Dan Cui, Dongyang Li, Runsheng Jiao, Xiaoran Wang, Shuwei Jia, Dan Hou, Tong Li, Haitao Liu, Ping Wang, Yu-Feng Wang

**Affiliations:** ^1^Department of Physiology, School of Basic Medical Sciences, Harbin Medical University, Harbin, China; ^2^Department of Genetics, School of Basic Medical Sciences, Harbin Medical University, Harbin, China

**Keywords:** intranasal drug delivery, olfactory bulbs, oxytocin, social preference, supraoptic nucleus

## Abstract

Social functions of oxytocin (OT) have been explored extensively; however, relationship between the effect of intranasally applied OT (nasal OT) on the social preference (SP) and intracerebral actions of endogenous OT remains unclear. To resolve this question, we first observed effects of nasal OT on the SP of virgin young adult male rats toward unfamiliar virgin estrous female (EF) vs. virgin male rats. The results showed that the test male rats exhibited significantly more times and longer duration accessing the female than the male, which were acutely eliminated by nasal OT. Then, we examined the approaches mediating nasal OT effects on the activity of potential brain targets in Western blots and found that nasal OT activated the olfactory bulbs (OBs) and the supraoptic nucleus (SON), but not the piriform cortex, amygdala and hippocampus as shown by significant changes in the expression of c-Fos and/or phosphorylated extracellular signal-regulated protein kinase (pERK) 1/2. Moreover, microinjection of TTX into the OBs blocked nasal OT-evoked increases in pERK1/2 levels as well as the molecular association between ERK1/2 and OT-neurophysin in the SON. Electrolytic lesions of the lateral olfactory tract did not significantly change the basal levels of pERK 1/2 in the SON; however, upon nasal OT, pERK 1/2 levels in the SON reduced significantly. Lastly, microinjection of L-aminoadipic acid (gliotoxin) into the SON to reduce OT levels reduced the duration of the test male’s accessing the EF and blocked the nasal OT-evoked increase in the duration of test male’s accessing the male while significantly increasing pERK1/2 levels in the amygdala. These findings reveal for the first time that nasal OT acutely eliminates virgin males’ SP to EFs via the OB-SON route and that OT neurons could mediate the social effects of nasal OT by suppressing social phobia generated in the amygdala.

## Introduction

Intranasally applied substances can get into the brain through an incomplete blood-brain barrier and then modulate social behaviors directly (Johnson and Young, 2017). However, some substances can modulate brain functions without significantly penetrating into the brain, as exemplified by oxytocin (OT; Leng and Ludwig, 2016). OT is a nonaneuropeptide mainly produced in the supraoptic nucleus (SON) and paraventricular nucleus in the hypothalamus (Hou et al., 2016). The pro-social effects of intranasally-applied OT (nasal OT, in brief) are likely involved in the activation of OT neurons by the mediation of the olfactory bulbs (OBs) as evidenced by the following facts. (1) Nasally-applied substances are usually accumulated in the OBs before entering other brain areas (Dhuria et al., 2010). (2) The OBs and/or their presynaptic neurons express not only odorant receptors but also many peptide receptors including OT receptors (OTRs; Meddle et al., 2007). (3) Outputs from the OBs can activate the SON via neural connections of the lateral olfactory tract (LOT; Smithson et al., 1989). (4) The SON has extensive neural connections with other brain areas (Hou et al., 2016) including piriform cortex (PC), amygdala, and hippocampus that can regulate social behaviors (Takayanagi et al., 2017). Thus, it is possible for nasal OT to exert its prosocial effects via the OB-SON approach. However, direct evidence supporting such a functional connection remains to be established.

Among many social functions, OT has been implicated in the social preference (SP), particularly the interest to access the sex partner of opposite sex (Behnia et al., 2014). However, different from the pair-bonding promotion effect to the sex partner, OT exerts an effect to remove SP toward (non-partner) opposite sex. As reported, nasal OT can make a married man keep a much greater distance between himself and an attractive woman but not men during the first encounter (Scheele et al., 2012). OT was also found to switch test males’ opposite sex preference to the same sex preference during cohabitation in rats; this effect is likely involved in the sexually dimorphic brain nuclei including the preoptic area and the SON (Triana-Del Rio et al., 2015). Thus, OT could play a key role in determining sex-associated SP. However, it remains to be examined what are the regulation of nasal OT effect on the SP of virgin males toward different sexes, the brain targets and the roles of the OB-SON approach in this nasal OT effect.

To address these questions, we used a rat model, first confirmed the SP of virgin male rats toward estrous female (EF) relative to male rats, and verified that nasal OT could remove, even reverse such a preference. Then, we examined effects of nasal OT on the activity of potential brain targets. The results revealed that nasal OT increased the activity of the OBs and SON. Further observations verified that electrolytic lesion of the LOT reversely reduced the activity of the SON in response to nasal OT, indicating that this pathway delivers excitatory signals to the SON, which could counterbalance inhibitory signals through other approaches from the OBs to the brain. Moreover, disruption of the structural integrity of the SON by intranuclear injection of L-aminoadipic acid (L-AAA, a gliotoxin) known to disrupt the functions of OT neurons in the SON (Wang and Hatton, 2009), increased the levels of activation of amygdala while blocking the reversal effect of nasal OT on male preference. This study reveals for the first time that the OB-SON-amygdala pathway mediates nasal OT-modulated SP of male rats.

## Materials and Methods

Adult virgin Sprague-Dawley rats (150–200 g, 50–60 days old) of both sexes, housed in 12:12-h light-dark cycle with freely accessing food and water before the study. The protocols used were in accordance with NIH Guidelines for the Care and Use of Animals and approved by the Animal Care and Use Committees of the Harbin Medical University.

### Social Preference Testing

**Figure 1:**
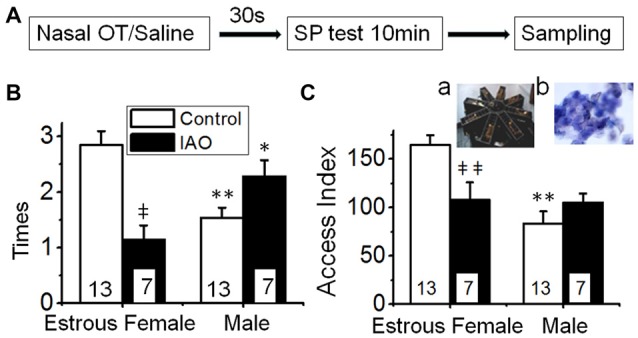
Intranasally-applied oxytocin (OT) removes test virgin males’ social preference (SP) to novel virgin estrous female (EF) vs. virgin male rats. **(A)** The flowchart of this study. **(B,C)** show the frequency (times/10 min) and access index (total duration) of the male rats’ accessing females vs. males in response to intranasal application of saline (open bar) or 0.1 nM OT (IAO, solid bar), respectively. The insets are a photo of the 8-arm radial maze **(a)** and a microscopic image of the vaginal smear of an EF rat **(b)**; numbers in the bars are that of rats; *P < 0.05 and **P < 0.01 compared to female group; ^‡^*P* < 0.05 and ^‡‡^*P* < 0.01 compared to control group.

A male rat was randomly selected as test rat and placed in the center of a modified 8-arm radial maze that had been installed a 6 × 15 × 20 cm^3^ cage (Figure 1, inset a) at the distal end of individual arms to hold the stimulus rats. In each test, one female and one male rats were randomly housed in two separate arms; they had approximately the same age and body weight. The time when the behavioral test was performed was 1–4 pm of the day after verification of the stage in estrous cycle of the female rats by vaginal smear in the morning (Figure 1, inset b). After a short period of adaptation for 10 min, the test male was allowed to enter any of the 8-arms freely. During the observation, the frequency (times in 10 min) and duration of the test male’s accessing different arms were recorded with a computer program (RM-200 Eight arm maze analysis software, Chengdu Techman Software Co. Ltd) and the data were used for late analysis of the SP. At the same time, the track of rat’s movements was also videotaped for verifying accuracy of the analyses. To avoid potential influence of spatial memory or the smells left by the preceding stimulus rats, stimulus rats were randomly assigned to different arms before the test and all arms were cleaned and aired thoroughly between tests.

### Brain Lesion and Drug Application

To block neural connections selectively through the LOT, electrolytic lesions were made at the LOT via dorsal approach. Under the anesthesia of 10% chloral hydrate (0.3 ml/100 g, i.p.), test male rats were placed in a stereotaxic frame according to a rat brain atlas (Paxinos and Watson, 1986). All incision areas and instruments were treated sterilely and 1% lidocaine was subcutaneously applied to avoid infection and minimize pain, respectively. Two holes on the bilateral frontal bone were drilled at 1.7 mm anterior to the Bregma point and 4.0 mm lateral to the midline and then a concentric stimulating electrode was vertically implanted through the holes with the tip at 7.8–8.0 mm below skull surface. A constant current (1.0 mA) was passed for 10 s for lesion of the two sites with 10 min interval. Then, the electrodes were removed and the skin was closed sterilely. The locations of the lesions were confirmed by microscopic identification of the electrode track and burning sign in *post hoc* histological examination 4 days later.

To determine if the SON was involved in the effect of nasal OT, microinjection of L-AAA into the SON was performed. Under the anesthesia of 10% chloral hydrate (0.3 ml/100 g), vehicle or 2 mM L-AAA in 2 μl artificial cerebrospinal fluid (aCSF) containing 0.4% Trypan blue was injected into the SON bilaterally according to the atlas (1.0 mm posterior to Bregma, 1.4 mm lateral to the midline and 9.5 mm below skull surface). These rats were used for testing SP 4 days later after full recovery from the surgical stress. The same surgeries were performed in the control groups except that L-AAA was replaced with aCSF. The actual injection site was verified by *post hoc* examination of the deposition of Trypan blue. All drugs were from Sigma-Andrich (Shanghai).

In testing effects of blocking neural conduction through the OBs on nasal OT-evoked changes in SON activity, aCSF containing 2 μM TTX (0.5 μl) or aCSF (vehicle) only were injected into bilateral OBs at a rate of 0.05 nl/min using a Hamilton microsyringe through holes drilled on the frontal bones (7.0 mm anterior to Bregma, 1.0 mm lateral and 5.0 mm below skull surface). Thirty minutes later, rats were prepared for examining effects of nasal OT on pERK1/2 expression in the SON.

### Nasal OT

In nasal drug application, rats were restrained in supine position and two drops of nasal saline (0.45% NaCl) or OT-containing nasal saline (0.1 nM, in 10 μl) were applied into each naris alternately. After the nasal treatment, rats were either used for testing SP at 30 s later or decapitated 10 min later for dissecting the hypothalamus for further processing.

### Western Blots and Co-Immunoprecipitation

After the SP tests, rats were decapitated and the hypothalamus were removed and cooled down in ice-cold aCSF for 1−2 min. The SON, main OBs, amygdala, PC, and hippocampus were punched-out of brain and homogenized in lysis buffer. Methods for processing the proteins were modified from that previously described (Wang et al., 2013a,b). In brief, 60 μg of protein per lane was separated on 10% SDS-PAGE gels and then transferred onto polyvinylidene difluoride membrane. Membranes were incubated with PBS containing 5% dry milk (w/v) for 1 h at 21–23°C and then incubated with antibodies (Santa Cruz Biotechnology, Shanghai) against glial fibrillary acidic protein (GFAP), OT-neurophysin (NP), c-Fos, total extracellular signal-regulated protein kinase (tERK or ERK) 1/2, phosphorylated extracellular signal-regulated protein kinase (pERK) 1/2, OTRs or Actin in a dilution of 1:300 at 4°C for 4 h (except for pERK1/2, 1:1000 dilution, and overnight). Actin, tERK 1/2 or tubulin was used as loading controls. The protein membranes were further processed with horseradish peroxidase-conjugated secondary antibodies and an enhanced chemiluminescence detection kit. Protein bands were visualized with an automated chemiluminescence imaging analysis system (Tanon 5200, Shanghai).

For co-immunoprecipitation, total lysate of the SON was cleared with protein A/G agarose (Millipore) and then 1.0–1.5 mg protein was incubated with 1.5 μg goat anti-tERK1/2 overnight at 4°C, to form an immune complex. The complex was captured by adding 50 μl of protein A/G agarose bead slurry and gently rocking for 2 h at 4°C. The complex-loaded beads were then collected by pulse centrifugation, washed, re-suspended in sample buffer and boiled for 10 min to separate and denature the protein complexes. The beads were then spun down, and the supernatant containing the denatured proteins was separated on 10% SDS-PAGE gels, transferred onto polyvinylidene difluoride membrane, and Western blots of OT-NP were performed to visualize individual bands. Positive and negative controls consisted of total lysate and non-specific IgG, respectively.

### Immunohistochemistry

In verification of the effect of L-AAA on astrocytic plasticity and OT neuronal activity in the SON, immunostaining was performed using the methods previously described (Wang et al., 2013a,b). In brief, the hypothalamus was fixed with 4% paraformaldehyde overnight, and then cut into 200 μm thick sections that contained the SON. The sections were treated with 0.3% Triton X-100 for 60 min to permeabilize the plasma membrane and then 0.3% gelatin for 60 min to block non-specific binding sites for the antibodies, respectively. After incubation with primary antibodies against GFAP, pERK 1/2 or OT-NP in a dilution of 1:300 at 4°C overnight, species-matched secondary antibodies (1:1000) were applied for 1.5 h to label the corresponding primary antibodies. Lastly, Hoechst stain (0.5 μg/ml, 15 min) was used to label nuclei. Sections were examined with a fluorescence microscope (Eclipse FN1, Nikon) through a CCD camera (DS Ri2, Nikon) or a confocal microscope (Thorlabs). To avoid false positive or negative results of immunostaining, serial dilutions of the primary antibody, staining with pre-absorbed (immune-neutralization) primary antibody, no-primary and no secondary antibody controls were applied.

### Data Analysis

The times traveling through the full length of an arm (frequency) and accumulative duration accessing different arms within 10 min were counted. To distinguish the total duration from individual access duration, we used the term of “access index” to indicate the accumulative/total duration of the test male in the arm with the stimulus female or male. The access index could better reflect the interest of rats toward their conspecifics or the corresponding clues than the average duration of each access that is obviously influenced by the frequency (times/10 min) of accessing.

Data were plotted as the mean ± SEM or the percentage of (loading) controls in Western blots, with “n” equal to the number of rats tested. Before determining statistical significance of different data sets, data distribution patterns and the homogeneity of variance were examined by use of Kolmogorov-Smirnov method and Levene method, respectively (SigmaPlot 11, Systat Software). Statistical significance (*P* < 0.05) was determined by *t*-test, chi-square test or ANOVA with the appropriate *post hoc* comparisons with Bonferroni correction as reported in “Results” section. In analyzing data of non-normal distribution or non-homogeneity of variance, Mann-Whitney U test or Correct *T* test were used when comparison was performed between two groups; Kruskal-Wallis method was carried out when three or more groups were treated. In the latter case, variations of statistical significance were further subjected to *post hoc* pairwise analysis by applying the Tamhane’s T2 method.

## Results

In this study, we first tested the preference of virgin males to their conspecifics and their SP toward different sexes, identified effects of nasal OT on the SP and the brain targets, established the link between the OBs and SON, and then confirmed the essential role of the SON in the nasal OT effects and its involvement of amygdala.

### Nasal OT Removes Social Preference of the Test Male to the Female vs. Male Rats

To verify the suitability using the 8-arm maze to detect the SP of the test rats, we first examined the frequency that the test male rats accessed the arm with or without a rat. The result showed that the frequency of the test male rats’ accessing the arms with rats (12.4 ± 0.7 times) was significantly higher (*n* = 12, *t* = 4.393, *P* < 0.01 by non-paired *t*-test) than that accessing the blank arms (8.2 ± 0.6 times). After nasal application of saline, the frequency of the test male rats’ accessing the arms with rats (16.2 ± 2.3 times) remained significantly higher (*n* = 6, *t* = 4.174, *P* < 0.01, by non-paired *t*-test) than that accessing the blank arms (6.3 ± 0.5 times). There is no significant difference (*t* = 1.947, *P* > 0.05 by Chi-square test) in the incidence accessing the arms with rats with and without prior application of nasal saline. This trend did not change significantly after nasal OT (16.1 ± 1.8 times with rats vs. 6.8 ± 0.4 times in blank, *n* = 7, *t* = 5.077, *P* < 0.01). These results support the suitability of using this modified 8-arm maze to examine SP and using nasal saline as a solvent to test drug effects.

Based on the findings presented above, we observed the frequency and duration of the test male rats’ accessing the female vs. male rats in the procedure shown in Figure 1A. The result showed that test males exhibited significant preference of accessing sexually receptive female than accessing the male as indicated by the frequency (2.8 ± 0.2 times to the female vs. 1.5 ± 0.2 times to the male rats, *n* = 13, *t* = 4.228, *P* < 0.01 by non-paired *t*-test) and access index (164.6 ± 10.1 s to the female vs. 83.3 ± 12.7 s to male rats, *n* = 13, *t* = 5.001, *P* < 0.01).

Upon nasal OT (0.1 nM, 10 μl for each naris) but not saline, the SP of test males toward EFs was removed. That is, nasal OT significantly increased the frequency accessing the stimulus males (2.3 ± 0.3 times with OT vs. 1.5 ± 0.2 times in the control, *n* = 7, *t* = −2.954, *P* < 0.05 by non-paired *t*-test) although it did not increase the access index significantly (105.3 ± 9.3 s vs. 83.3 ± 12.7 s in the control, *n* = 7, *t* = −1.171, *P* > 0.05). In contrast, nasal OT significantly decreased the preference toward the female as shown in the frequency (1.1 ± 0.3, *n* = 7, *t* = 4.351, *P* < 0.01 compared to 2.9 ± 0.3 in the control) and access index (107.9 ± 18.4 s, *n* = 7, *t* = 2.955, *P* < 0.01 compared to 164.6 ± 10.1 s in the control). Figures 1B,C are the summary of effects of nasal OT on the SP of the test male rats.

### Nasal OT and Brain Activity

The SP is determined by the activities of brain regions that have been implicated in regulating sex steroid hormones, sex behavior, rewarding, social cognition and social memory including the PC, OBs, SON, amygdala and the hippocampus. Selection of the SON is because it is a major brain area containing OT neurons and expressing OTRs (Meddle et al., 2007), and is a key link of the olfaction-hypothalamic neuroendocrine connection (Hou et al., 2016). The OBs are the major relay of nasal drug which effects brain functions (Leng and Ludwig, 2016) including that of the SON (Hatton and Wang, 2008). The amygdala and PC are major targets of the olfactory system and have close interactions with the SON in social recognition (Chang et al., 2015). Selection of the hippocampus is not only because of its close interaction with the SON in social function but also because of the demand to excluding memory effect of the 8-arm radial maze that is a typical device for learning and memory studies.

**Figure 2:**
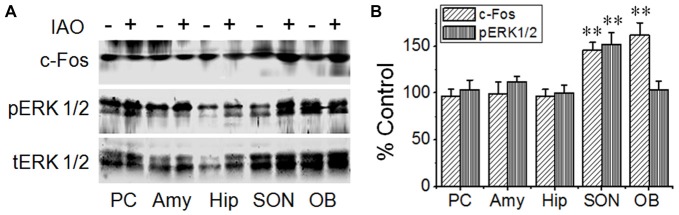
Effects of nasal OT on phosphorylated extracellular signal regulated protein kinase (pERK) 1/2 and c-Fos expressions in different brain regions. **(A)** Exemplary protein bands showing the expressions of c-Fos (top panel), pERK 1/2 (middle panel) and total (t)ERK 1/2 (bottom panel), respectively from the piriform cortex (PC), amygdale (Amy), hippocampus (Hip), supraoptic nucleus (SON) and main olfactory bulb (OB). **(B)** Bar graph summarizing the effect of nasal OT on the expressions of pERK 1/2 and c-Fos at different brain areas; **P < 0.01 compared to the control group. Other annotations refer to Figure 1.

Here, we first evaluated effects of nasal OT on the activity of these brain regions using c-Fos protein and pERK 1/2 as biomarkers for cellular activation in Western blots (Figure 2). At 10 min after nasal OT, significant increases in c-Fos and/or pERK 1/2 levels occurred in the SON (c-Fos: 146.2 ± 8.1% of control, *n* = 9, *t* = −5.7, *P* < 0.01; pERK1/2: 152.4 ± 12.8% of control, *n* = 9, *t* = −4.1, *P* < 0.01) and the OBs (c-Fos: 162.0 ± 13.4% of control, *n* = 9, *t* = −4.6, *P* < 0.01; pERK1/2: 103.0 ± 9.2% of control, *n* = 9, *P* > 0.05), but not in the PC (96.5 ± 7.5% of control c-Fos, *n* = 9, *t* = 0.47, *P* > 0.05; 103.7 ± 10.2% of control pERK1/2, *n* = 4, *t* = −0.36, *P* > 0.05), amygdala (99.4 ± 12.5% of control c-Fos, *n* = 9, *t* = 0.05, *P* > 0.05; 111.6 ± 6.4% of control pERK1/2, *n* = 9, *t* = −1.82, *P* > 0.05) and hippocampus (96.5 ± 7.3% of control c-Fos, *n* = 9, *t* = 0.48, *P* > 0.05; 99.8 ± 8.6% of control pERK1/2, *n* = 9, *t* = 0.02, *P* > 0.05). The results are in agreement with the proposal that the removing effect of nasal OT is likely mediated by the OB-SON approach.

### OB Mediation of Nasal OT-Elicited Activation of the SON Via the LOT

**Figure 3:**
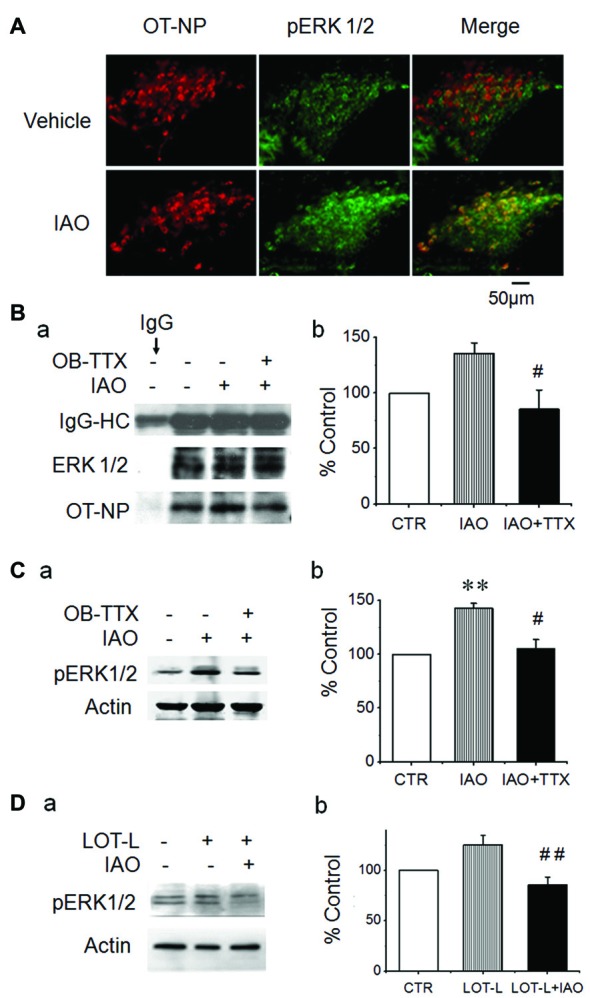
Effects of blocking neural conduction in the OBs and through the lateral olfactory tract (LOT) on nasal OT-elicited pERK 1/2 expressions in the SON. **(A)** Microscopic images of nasal OT-evoked pERK 1/2 expression in the SON showing (from left to the right) OT-neurophysin (NP), pERK 1/2 and their merge, respectively. **(B)** Exemplary protein bands **(Ba)** showing result of co-immunoprecipitation of ERK 1/2 (middle) with OT-NP (bottom) following nasal OT without and with prior intra-OB application of TTX and their summary **(Bb)**. IgG-HC, the heavy chain of immunoglobulin G. **(C)** Effects of pretreatment of the main OBs with vehicle (Veh., artificial cerebrospinal fluid, aCSF) or TTX (2 μM, 0.5 μl) on nasal OT-evoked pERK 1/2 expressions. Exemplary Western blot bands **(Ca)** and average levels (**Cb**, % relative to 10 min of 0.45% saline) of pERK 1/2 in the SON 10 min after nasal OT in rats. **(D)** Effects of electrolytic lesion of the LOT (LOT-L) on nasal OT-evoked pERK 1/2 expressions. **(Da)** Exemplary Western blot bands. **(Db)** Bar graph summarizing the effect of nasal OT without and with LOT lesions based on six rats in each group. **P < 0.01 compared to the control group; ^#^*P* < 0.05; ^##^*P* < 0.01 compared to IAO or the LOT lesion group. Other annotations refer to Figures 1, 2.

The significant activation of the OBs and the SON but not the PC suggests that there is some inherent association between OBs and the activation of the SON in nasal OT-elicited SP change. To validate this hypothesis, we observed the effect of nasal OT on pERK 1/2 levels in the SON after application of TTX into the central area of the main OBs. The results showed that nasal OT significantly increased the colocalization of OT-NP with pERK 1/2 in immunohistochemistry (Figure 3A) and co-immunoprecipitation exhibited a reduction of molecular association of tERK 1/2 with OT-NP after intra-OB application of TTX (85.5 ± 17.3% of the control, *n* = 3, *t* = 5.25, *P* < 0.05; Figure 3B). Further quantitative study in Western blot (Figure 3C) showed that nasal OT evoked significant increases in pERK 1/2 levels in the SON (143.2 ± 4.4% of the control, *n* = 5, ANOVA, *F* = 6.81, *P* < 0.01) and pre-treatment with TTX significantly reduced nasal OT-evoked pERK 1/2 increase in the SON (105.1 ± 8.6% of the control, *n* = 5, *P* > 0.05 compared to the control and *P* < 0.05 compared to nasal OT alone). This result is in agreement with the excitatory output from the OBs to the SON (Hatton and Wang, 2008).

To identify the potential link between the OBs and SON, electrolytic lesions were made at the LOT and then the effect of nasal OT on pERK 1/2 expression in the SON was examined (Figure 3D). The result showed that electrolytic lesions of the LOT did not significantly change the basal levels of pERK 1/2 (125.2 ± 9.6% of the control, *n* = 6, *P* > 0.05); however, upon nasal OT, pERK 1/2 levels in the SON reduced significantly compared to the rats with LOT lesion only (85.3 ± 8.0% of the control, *n* = 8, ANOVA, *F* = 6.43, *P* < 0.01).

### Involvement of the SON in OT-Evoked Changes in the Social Preference

To examine if the SON is a major mediator of the nasal OT-evoked SP change (Triana-Del Rio et al., 2015), we observed effects of disturbing cellular activities in the SON on nasal OT-evoked SP change 4 days after microinjection of either aCSF or L-AAA into the SON.

**Figure 4:**
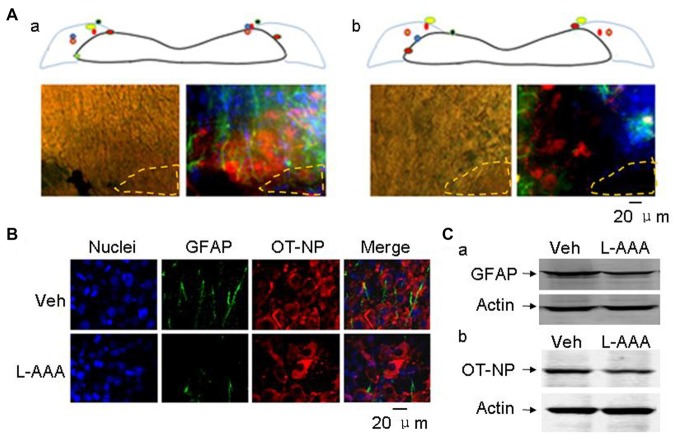
Effects of l-aminoadipic acid (L-AAA) on the expression of glial fibrillary acidic protein (GFAP) and OT-NP in the SON. **(A)** Central loci (upper panels, marked with colored dots) of intra-SON microinjection of aCSF (**Aa**, 2 μl) and L-AAA (**Ab**, 2 mM, 2 μl) containing 0.4% Trypan blue and their effects on GFAP and OT-NP in microscopic immunostaining images (lower panels), respectively. The images are present in bright field (left) and in merged images of nuclei (in blue), GFAP (in green) and OT-NP (in red). **(B)** Microscopic images showing immunostaining of nuclei (in blue), GFAP (in green), OT-NP (in red) and their merges without (top panels, vehicle, veh) and with (bottom panels) L-AAA treatment. **(C)** Western blotting bands showing GFAP **(Ca)** and OT-NP **(Cb)** expression in the SON without and with intranuclear L-AAA injection. Other annotations refer to Figure 3.

In seven test male rats with bilateral injection of aCSF, five showed clear labeling of bilateral SON in the middle coronal sections and the SON in other two rats was partially labeled. In the immunostaining, there was no clear Trypan blue labeling of the SON cells and the distribution of GFAP and OT-NP remained in normal patterns (Figure 4Aa). In six test male rats with bilateral microinjection of L-AAA, four rats showed clear labeling in the middle coronal sections of bilateral SON and the SON in other two rats were partially marked (Figure 4Ab). Histologically, the majority of SON cells marked with Trypan blue were either shrunken or swollen while GFAP, a major indicator of OT neurons-associated astrocytic plasticity (Wang and Hamilton, 2009), disappeared or reduced in the dye-labeled area and OT-NP positive staining also reduced 4 days after L-AAA treatment (Figure 4B). In Western blots, protein levels of both GFAP (50.1 ± 7.5% of vehicle, *n* = 7, *t* = 6.66, *P* < 0.01, Figure 4Ca) and OT-NP (66.2 ± 12.9% of vehicle, *n* = 7, *t* = 6.62, *P* < 0.05, Figure 4Cb) reduced significantly compared to the vehicle group. These findings are consistent with the effect of L-AAA on GFAP and OT neuronal activity in the SON reported previously in lactating rats (Wang and Hatton, 2009).

**Figure 5:**
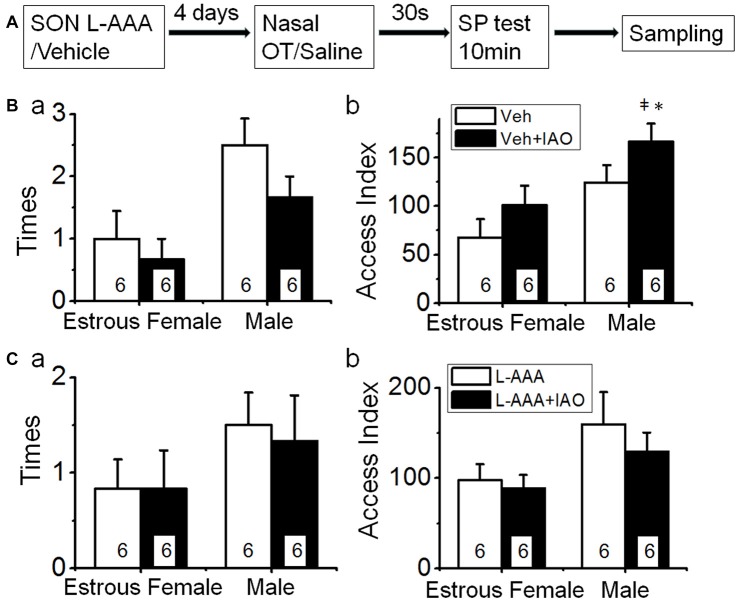
Effects of disturbance of supraoptic integrity on nasal OT-changed SP. **(A)** The flowchart of this study. **(B)** Mechanical disturbing the SON with aCSF on nasal OT-evoked switch of SP. **(Ba,Bb)** The times **(a)** and access index **(b)** of the male rats’ accessing the female vs. the male after nasal application of saline (open bar) or 0.1 nM OT (solid bar) at 4 days after intra-SON application of aCSF, respectively. **(C)** Influence of disruption of the SON by intranuclear application of L-AAA on nasal OT-evoked switch of SP. **(Ca,Cb)** are the same as **(Ba,Bb)**. *P < 0.05 and **P < 0.01 compared to female group; ^‡^*P* < 0.05 and ^‡‡^*P* < 0.01 compared to control group. Other annotations refer to Figures 1, 4.

After directly applying aCSF into the SON, behavioral tests (Figure 5A) showed that the basal frequency of the test male’s accessing the female in the vehicle group (1.0 ± 0.4, *n* = 6) was significantly lower (*t* = 3.61, *P* < 0.01) than that in the control that did not receive intra-SON microinjection (2.8 ± 0.2 times), and the reversal effect of nasal OT on the accessing frequency was weakened (0.7 ± 0.3, *n* = 6, *t* = 0.60, *P* > 0.05 compared to the vehicle). The access index to the female (67.7 ± 18.8) was also significantly lower (*n* = 6, *t* = 4.96, *P* < 0.01) than that of control (164.6 ± 10.1 s). This finding is in agreement with the view that structural integrity of the SON is critical in brain social function (Hou et al., 2016). Following nasal OT, the access index to the female did not change significantly (101.0 ± 14.8 s, *n* = 6, *t* = −2.46, *P* > 0.05 compared to the vehicle).

By contrast, the basal frequency accessing the male (2.5 ± 0.4 times, in vehicle, *n* = 6) was significantly higher than that in the control condition that did not receive intra-SON microinjection (1.6 ± 0.2 times, *t* = −2.45, *P* < 0.05) but had no difference from that after nasal OT (1.7 ± 0.3 times, *n* = 6, *t* = 1.39, *P* > 0.05). Similarly, the basal access index (124.3 ± 17.8 s, *n* = 6) to the male did not increase significantly (*t* = −1.88, *P* > 0.05) compared to that in the control condition (83.3 ± 12.7 s). Following nasal OT, the access index to the male (166.2 ± 18.6 s, *n* = 6, *t* = −3.61, *P* < 0.05) further increased compared to the basal condition. These results indicate that nasal OT plays a more specific role in increasing the SP toward a male conspecifics (Figure 5B).

After L-AAA treatment (Figure 5C), the frequency of accessing the female (0.8 ± 0.3 times, *n* = 6) was significantly lower (*t* = 4.75, *P* < 0.01) than that in the control and was not significantly influenced by nasal OT (0.8 ± 0.4 times, *n* = 6, *t* = 0, *P* > 0.05). The access index to the females (97.7 ± 17.8, *n* = 6) was significantly lower (*t* = 3.50, *P* < 0.01) than that of the control and nasal OT failed to change the access index significantly (88.7 ± 14.8 s, *n* = 6, *t* = 0.42, *P* > 0.05). Moreover, the frequency of test male’s accessing the male did not change significantly (1.5 ± 0.3 times in L-AAA vs. 1.3 ± 0.8 times in L-AAA + OT, *n* = 6, *t* = 0.35, *P* > 0.05); however, nasal OT lost its reversal effect on the access index to the male (159.5 ± 35.7 s in L-AAA vs. 129.3 ± 21.1 s in L-AAA + OT, *n* = 6, *t* = 0.37, *P* > 0.05). These results are consistent with the critical role of astrocyte integrity in the functions of SON neurons (Wang and Hatton, 2009) and the potential involvement of the OT-secreting system in the regulation of SP (Triana-Del Rio et al., 2015), specifically to nasal OT-evoked male preference.

### SON Modulation of Nasal OT-Evoked Activation of the Amygdala

**Figure 6:**
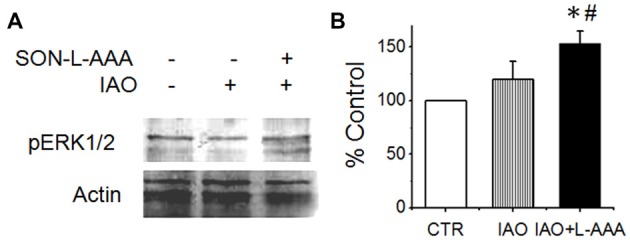
Effects of disrupting the SON on nasal OT modulation of the activity of amygdala. **(A,B)** Western blot bands **(A)** and levels **(B)** of pERK 1/2 vs. actin10 min after nasal application of saline (CTR) or OT (0.1 nM, right two lanes) with pretreatment of the SON with aCSF (left two lanes) or L-AAA (the right lane), respectively. *P < 0.05 compared to the control group; ^#^*P* < 0.05 compared to IAO group. Other annotations refer to Figure 4.

To test if disturbing the activity of the SON would change the activity of amygdala that could contribute to social phobia (Amaral, 2003), we observed effects of nasal OT on pERK 1/2 expression in the amygdala after application of L-AAA into the SON. As shown in Figure 6, nasal OT did not significantly increase pERK 1/2 levels in the amygdala (119.9 ± 16.7% of the vehicles (*n* = 6, *P* > 0.05); however, after L-AAA pre-treatment, nasal OT significantly increased pERK 1/2 levels (152.9 ± 11.8% of the control, *n* = 6, *P* < 0.05) compared with control or nasal OT group (*F* = 5.9, *p* < 0.05 by ANOVA). This result is in agreement with the finding that OT could inhibit the activity of limbic amygdala through the GABAergic system (Knobloch et al., 2012).

## Discussion

The present studies revealed that nasal OT acutely eliminated the SP of test virgin male rats toward sexually receptive female vs. the stimulus male, and even switched the preference. Nasal OT activated the OBs, which led to SON activation mediated by the LOT. Moreover, after disruption of the SON, nasal OT reversely increased the activity of the amygdala, indicating a tonic inhibition of amygdala activity by nasal OT-evoked activation of the SON. Thus, the present study reveals for the first time that nasal OT can remove the SP of test males toward EFs over the males, which is mainly mediated by the OB-SON-amygdala pathway.

### Methodological Consideration

Sexual characteristics are usually indicated by sex organs and secondary sex characteristics including facial and body hair, size of breasts, estrous cycle, body form, relative height and body fat, etc. In addition, sound and smell as well as behaviors can reveal the biological sex characteristics of the organism, and thus become the extrinsic signs for an individual to identify their sex. To evaluate the sex-associated SP of males through these extrinsic signs, it is necessary to create an environment for a test male to perceive these features in a suitable environment. The modified 8-arm radial maze became our choice because it allows a rat to access blank arms or the arms with rats, either a female or a male, randomly. Relative to a two choice device, the modified 8-arm device provided multiple choices for the test male and could better reflect his SP. To eliminate the potential confounding effect of multiple blank arms in the test, we also tested males’ SP using a Y maze; the same findings as those in the 8-arm radial maze were observed (Supplementary Figure S1).

A further consideration is the memory effects that initially endows to the usage of the 8-arm maze. Without prior training for the spatial memory, the device would not exert its function of memory test, which is consistent with the negative result of nasal OT on hippocampal pERK1/2 expression in Western blots. Lastly, with the installation of a simple separation cage, the rats could communicate via sounds and smells without direct physical contacts. This can largely exclude the influence of female’s SP on the judgment of the test male’s SP, which could occur in an open field test device. Thus, this modified 8-arm radial maze was used for examining the SP of males.

### OT and Social Preference

In animals, the SP reflects the components of both sociality and reproduction demands observed in human beings (Behnia et al., 2014). By selectively accessing one sex, individuals can meet the requirement of merging into a society or achieving the goal of reproduction. Nevertheless, the SP is built on its own biological basis involving the genetic inheritance, physical maturation, sexual hormones, rewarding system, cognition, cultural/social background and many other factors (Govic et al., 2008; Pfaus et al., 2012). For reproduction, contacting sexually accessible females by adult males is necessary, which is again verified in the present study based on adolescent/virgin males. Previous study on the SP mainly focused on pair-bonding or cohabitation of prairie voles (Keebaugh et al., 2015; Pan et al., 2016); however, there is no report about acute switching effects of nasal OT on the SP of virgin male rats. In the present study, we confirmed previous finding that OT can switch the SP of the test male from female to male in human beings (Scheele et al., 2012) and in rats (Triana-Del Rio et al., 2015) while extending the roles of nasal OT on the SP into virgin males in an acute process.

Previous studies revealed that the SP depends on prior sexual experiences (Scheele et al., 2012) as well as the time and dose of OT applied (Smith et al., 2010; Bales et al., 2013). As described by Bales et al. (2013), the prairie vole treated with OT acutely increased social behavior in male voles with familiar partners, as seen in humans. However, long-term developmental treatment with low doses of intranasal OT resulted in a deficit in partner preference behavior by male voles. Thus, a male rat without sex experience prefers to access a sexually attractive female and the losses of preference or a switch of such a preference are not contradictory to these previous reports including the facilitatory effect on partner attachment of OT.

The present study further highlights an “anti-estrous pheromone effect” of nasal OT in virgin males. As shown in Supplementary Figure S2, nasal OT could also switch the SP of a test male from EF to non-EF. Moreover, nasal OT also removed the interest of a test male from the virginal smear of an EF to that of non-EF (Supplementary Figure S3). Thus, the nasal OT-evoked SP change of the test male is largely due to the “anti-estrous pheromone effect”, but not an aversion to the female. This finding also suggests that in the pair-bonding effect of nasal OT (Veening et al., 2015), the function of OT is not likely mediated by the estrous pheromone of the female partner but some other mechanisms that remain to be identified.

### Nasal OT-Evoked Social Preference Change by Mobilizing Endogenous OT Via OB-SON Route

Responsive OT releases into the circulation and the brain (Neumann et al., 1996) are determined by OT neuronal activity (Hatton and Wang, 2008). Our observation also revealed that nasal OT could increase OT neuronal activity as indicated by nasal OT-evoked increase in the co-localization (Figure 3A) and co-immunoprecipitation of OT-NP with ERK 1/2 in the SON (Figure 3B). Thus, nasal OT-evoked changes in male SP are related to the activation of OT neurons. This proposal is supported by the finding that L-AAA treatment in the SON reduced OT content while blocking nasal OT-evoked SP change and that sexually dimorphic brain nuclei including the preoptic area and the SON were involved in the SP (Triana-Del Rio et al., 2015).

Present study reveals that both structural and functional integrities of the SON are essential for the SP of male rats. Intra-SON application of aCSF blocked the natural predominant SP of the test rats to the EFs although it did not significantly influence the microscopic structures of the SON. Thus, functional integrity of the SON is the key to maintaining normal SP of the males. Moreover, that structural destruction of the SON with L-AAA blocked nasal OT-evoked SP change not only indicates the importance of structural integrity of the SON, but also affirms the importance of the functional integrity of the SON in the nasal OT effects.

GFAP is the basis of astrocytic plasticity (Wang and Parpura, 2016) and has critical influence on OT neuronal activity (Wang and Hatton, 2009). The reduction of GFAP and the accompanying OT decrease following L-AAA treatment highlight the importance of both SON integrity and the OT-secreting system in nasal OT-evoked SP change of the males. Noteworthy is that the maintenance of the SP toward EF is more sensitive to the integrity of the SON, relative to nasal OT evoked SP toward the male conspecifics.

Further analysis reveals that nasal OT-evoked SON activation is mediated through the OB-SON route. Among multiple approaches (Quintana et al., 2015) that could mediate this nasal OT effect on the SON, the OBs are the major one (Dhuria et al., 2010), which is supported by the present finding that the OBs express OTRs and nasal OT could increase the expression of c-Fos and pERK 1/2. Interestingly, the effect of nasal OT on the expression of c-Fos in the OBs and SON could occur in 10 min, and that is consistent with a previous finding (Willoughby et al., 1997). They together indicate that c-Fos expression can be increased significantly before its peak. Moreover, blocking neural conduction with TTX in the OBs also blocked nasal OT-evoked activation of the SON. In the neurotransmission from the OBs to the SON, the LOT plays a major mediator role. This proposal is supported by the effect of electrolytic lesions of the LOT on pERK 1/2 levels in the SON and by the known neural connections between the two structures (Hatton and Wang, 2008).

Noteworthy is that the present result cannot exclude the contribution of accessory OBs to the SP of the males toward the EFs and the effects of nasal OT. However, the blocking effects of intra-OB application of TTX on OT modulated SP strongly support a major contribution of the main OBs in this process. Together, we believe that nasal OT-evoked SP change of the male rats is mediated by the nose-brain route via the OBs-SON connection.

### Nasal OT-Evoked Changes in Social Preference by Inhibition of Amygdala Activity

Many brain regions have been implicated in the regulation of sex-associated social activities including the preoptic area (Pfaus and Heeb, 1997), the amygdala (Rupp et al., 2013) and the medial prefrontal cortex (Nakajima et al., 2014) in addition to the OBs (Kimchi et al., 2007) and the SON. These brain areas all possibly receive OTergic influences from the SON (Hou et al., 2016) directly or through the perinuclear zone neurons (Alonso et al., 1986) and CSF (Ju et al., 1986). Among them, amygdala is an important candidate for nasal OT-evoked SP change (Beery and Zucker, 2010). The activation of amygdala can inhibit social interaction with novel conspecifics and its activation is likely associated with social phobia (Amaral, 2003). Under natural condition, external signs of the female and estrous pheromone(s) are likely the major attractants for the virgin males, and could inspire the interests of test males to approach the respondents for further investigation. Nasal OT activates the SON likely by evoking a LOT-mediated dominant excitatory input and a weak inhibitory input on OT neurons in the SON since nasal OT decreased but did not increase pERK 1/2 expression after the LOT lesion. The output from the SON exerts an inhibitory effect on the activity of the amygdala through the GABAergic inhibitory system (Knobloch et al., 2012) and the SON lesion should weaken such inhibition, thereby allowing nasal OT to evoke excitation of the amygdala. Since activation of the amygdala can cause fear and social phobia (Amaral, 2003), the increased activity of the amygdala following nasal OT in SON lesion rats should intensify the fear of the test male toward other competent males, thereby making nasal OT-evoked SP change fail to occur.

## Concluding Remarks

**Figure 7:**
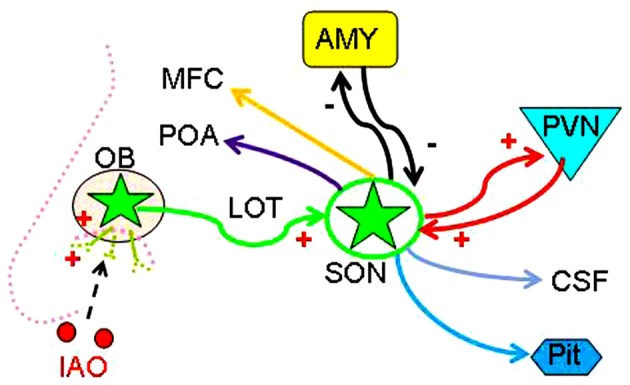
Hypothetical OB-SON-social brain pathway mediating nasal OT-evoked SP change of male rats. Abbreviations: CSF, cerebrospinal fluid; MFC, medial frontal cortex; POA, preoptic area; Pit, posterior pituitary; PVN, paraventricular nucleus. Other annotations refer to Figures 1, 2.

The present study provides the first report of functional involvement of supraoptic OT neurons in nasal OT-evoked SP change in adolescent/virgin males and outlines an OB-SON-social brain route (Figure 7). The major challenge in furthering this study is at clarifying mechanisms underlying nasal OT-evoked change of the SP, particularly for the different effects of nasal OT on the SP between female (Beery and Zucker, 2010) and male (Triana-Del Rio et al., 2015) and between virgin and sexually-experienced males. It is also a question that if social prairie voles use mechanisms different from other strains of rodents in the SP because central OTRs mediate mating-induced partner preferences and enhance correlated activation across forebrain nuclei in male prairie voles (Johnson et al., 2015), which is not reported in other strains. In addition, evaluation of the potential contribution of the accessory OBs in the effect of nasal OT is necessary to be performed. To answer these questions, expression levels of sexual steroid hormone receptors in the amygdala could be a clue since low levels of estrogen receptor-α in the medial amygdala are necessary to “permit” the expression of high levels of male prosocial behavior (Cushing et al., 2008). Moreover, sex-associated dopaminergic rewarding system is worth exploring because the SP is associated with brain rewarding system including the ventral tegmental area (Song et al., 2016) and the nucleus accumbens (Scheele et al., 2016) in addition to the central amygdala (Beery and Zucker, 2010). Further clarification of the underlying mechanism will profoundly influence our views of the mechanisms underlying effects of intranasal drugs and provide a novel model of studying on the brain social functions of other neuropeptides.

## Author Contributions

X-YL, DC and DL performed the experiments and analyses; X-YL wrote the first draft; PW and Y-FW designed the experiments; all participated in discussions and revisions of the article.

## Conflict of Interest Statement

The authors declare that the research was conducted in the absence of any commercial or financial relationships that could be construed as a potential conflict of interest.

## Supplementary Material

FIGURE S1 Estrous female (EF) preference of a test virgin male and its reversal by nasal OT in a Y maze. Note, ***P* < 0.01 compared to the blank arm (None); ^‡‡^*P* < 0.01 comparison between control and IAO; and ^##^*P* < 0.01 compared to estrous female group.

FIGURE S2 Nasal OT removes test virgin males’ social preference from estrous female to non-estrous female rats. **(A,B)** show the frequency and access index of the test male rats to estrous (EF) vs. non-estrous (NEF) females before (open bar) and after (solid bar) intranasal application of saline or OT, respectively. **P* < 0.05 and ***P* < 0.01 compared to EF group; ^‡^*P* < 0.05 and ^‡‡^*P* < 0.01 compared to control group. Annotations refer to Supplementary Figure S1.

FIGURE S3 Nasal OT removes test virgin males’ interest from the virginal smear of estrous female to the smear from non-estrous female rats. **(A,B)** show the frequency and access index of the male rats to estrous smear vs. non-estrous smear before (open bar) and after (solid bar) intranasal application of saline or OT, respectively. Note that the smear on the cotton ball was hid in the cage. **P* < 0.05 and ***P* < 0.01 compared to EF group; ^‡^*P* < 0.05 and ^‡‡^*P* < 0.01 compared to control group. Other annotations refer to Supplementary Figure S1.
