# Supplementary figures and images for: Oxytocin Removes Estrous Female vs. Male Preference of Virgin Male Rats: Mediation of the Supraoptic Nucleus Via Olfactory Bulbs
Source: Front Cell Neurosci. 2017 Oct 23;11:327. doi: 10.3389/fncel.2017.00327 (PMC5660071; doi:10.3389/fncel.2017.00327)

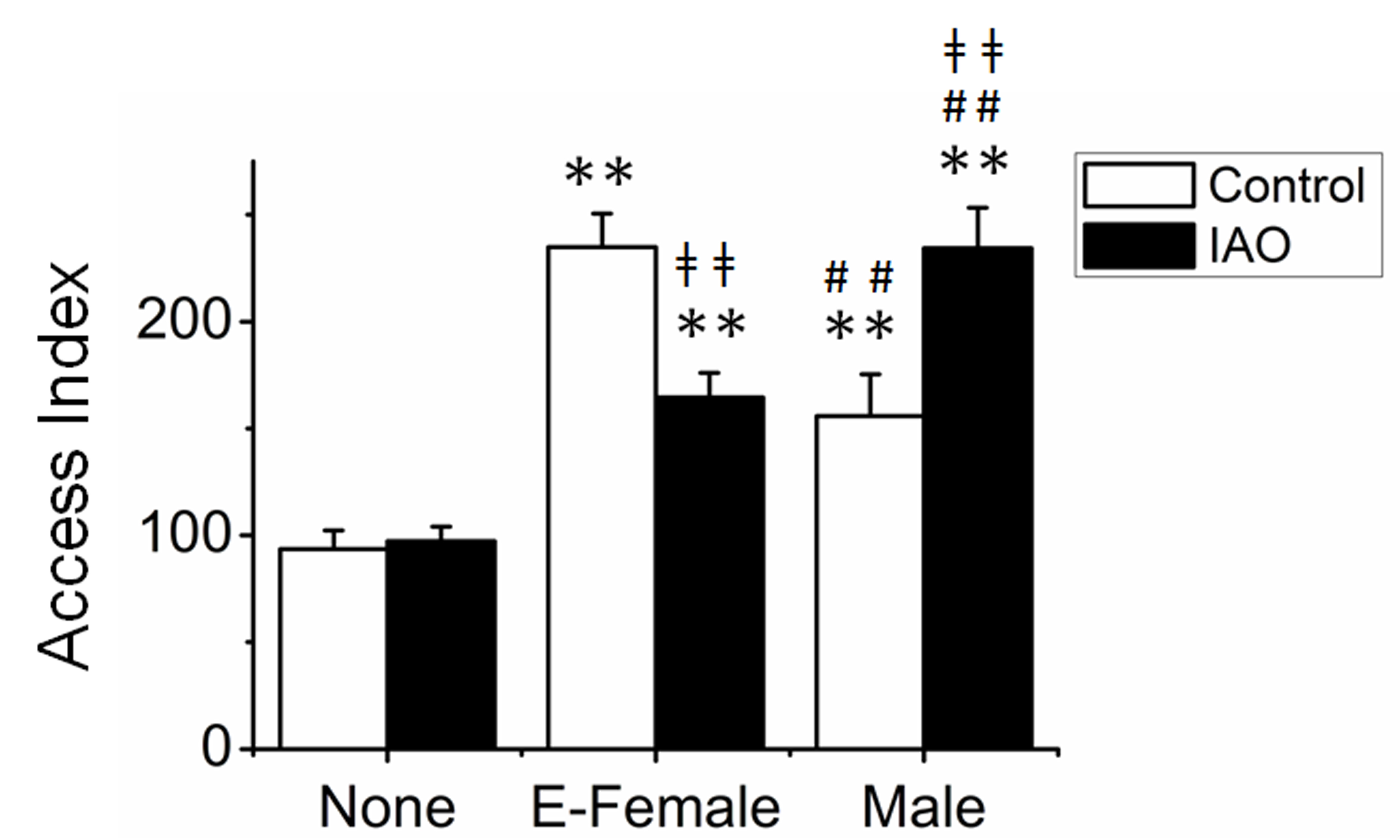

Supplement: FIGURE S1 — Estrous female (EF) preference of a test virgin male and its reversal by nasal OT in a Y maze. Note, **P < 0.01 compared to the blank arm (None); ‡‡P < 0.01 comparison between control and IAO; and ##P < 0.01 compared to estrous female group. [file Image_1.jpeg]

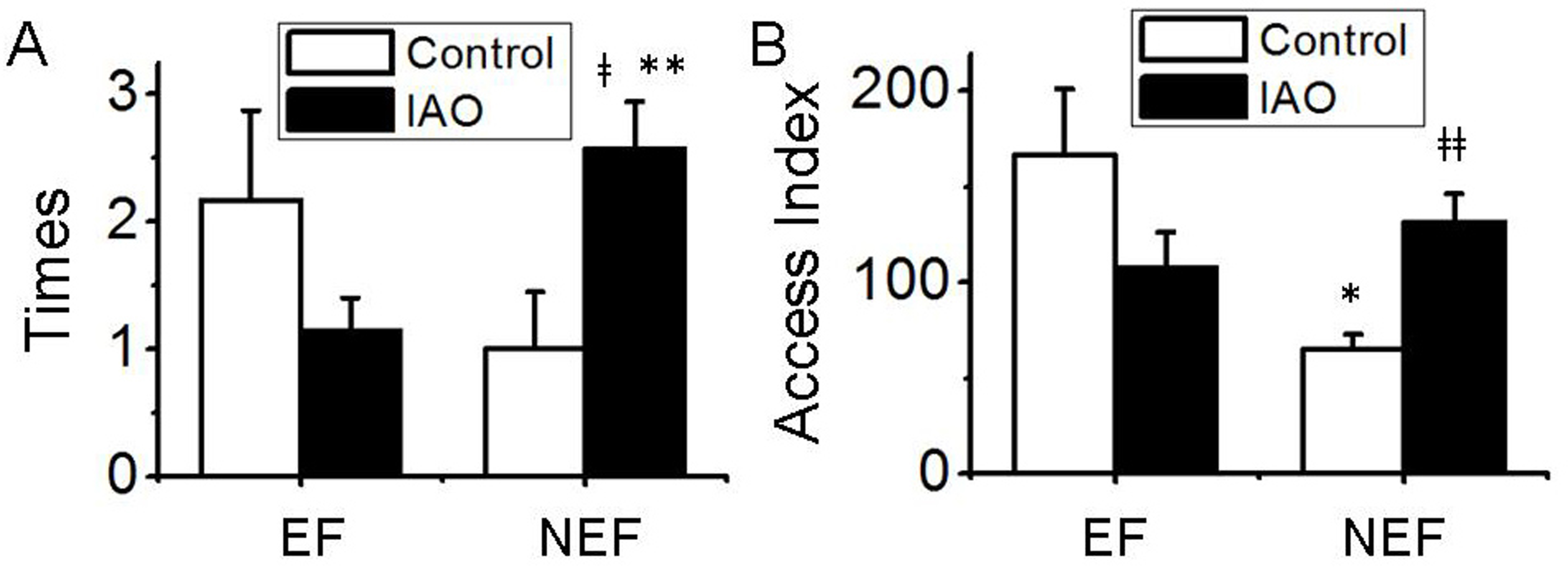

Supplement: FIGURE S2 — Nasal OT removes test virgin males’ social preference from estrous female to non-estrous female rats. (A,B) show the frequency and access index of the test male rats to estrous (EF) vs. non-estrous (NEF) females before (open bar) and after (solid bar) intranasal application of saline or OT, respectively. *P < 0.05 and **P < 0.01 compared to EF group; ‡P < 0.05 and ‡‡P < 0.01 compared to control group. Annotations refer to Supplementary Figure S1. [file Image_2.jpeg]

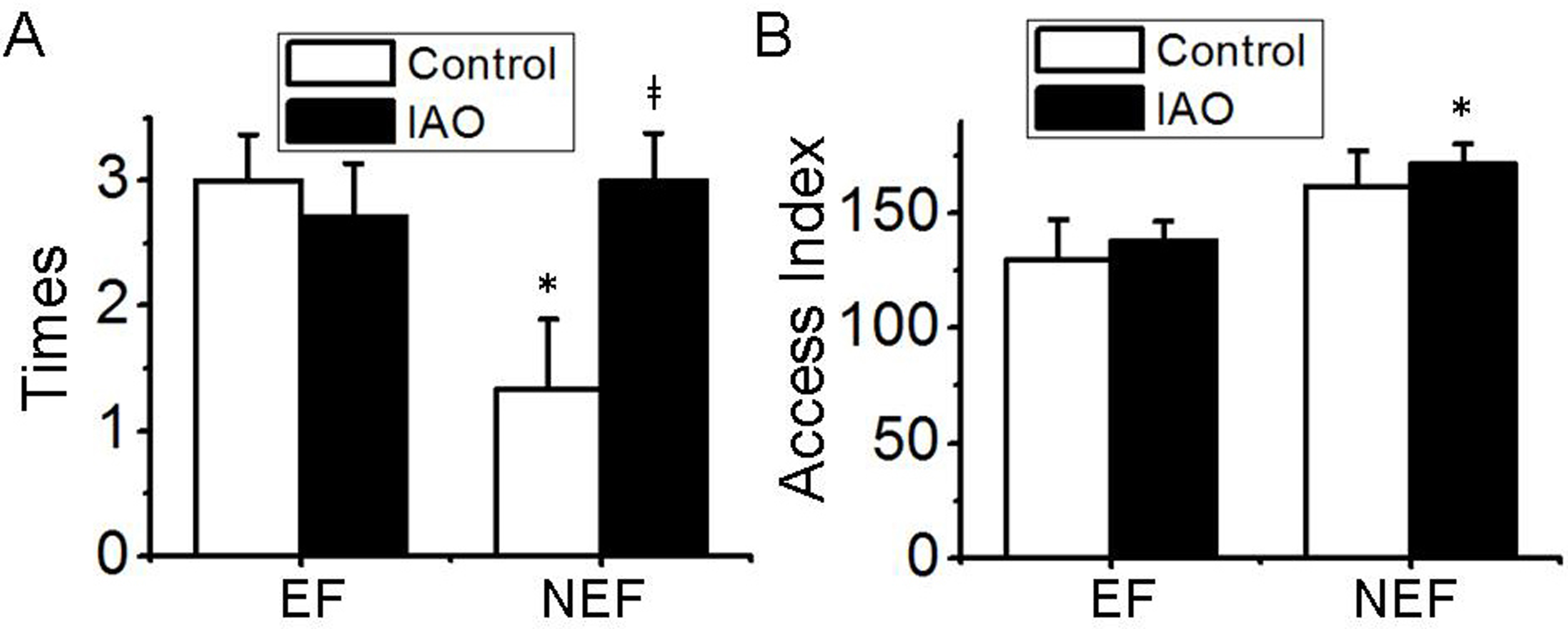

Supplement: FIGURE S3 — Nasal OT removes test virgin males’ interest from the virginal smear of estrous female to the smear from non-estrous female rats. (A,B) show the frequency and access index of the male rats to estrous smear vs. non-estrous smear before (open bar) and after (solid bar) intranasal application of saline or OT, respectively. Note that the smear on the cotton ball was hid in the cage. *P < 0.05 and **P < 0.01 compared to EF group; ‡P < 0.05 and ‡‡P < 0.01 compared to control group. Other annotations refer to Supplementary Figure S1. [file Image_3.jpeg]
